# Novel and Convenient Method to Evaluate the Character of Solitary Pulmonary Nodule-Comparison of Three Mathematical Prediction Models and Further Stratification of Risk Factors

**DOI:** 10.1371/journal.pone.0078271

**Published:** 2013-10-29

**Authors:** Fei Xiao, Deruo Liu, Yongqing Guo, Bin Shi, Zhiyi Song, Yanchu Tian, Chaoyang Liang

**Affiliations:** Department of Thoracic Surgery, China-Japan Friendship Hospital, Beijing, China; Roswell Park Cancer Institute, United States of America

## Abstract

**Objective:**

To study risk factors that affect the evaluation of malignancy in patients with solitary pulmonary nodules (SPN) and verify different predictive models for malignant probability of SPN.

**Methods:**

Retrospectively analyzed 107 cases of SPN with definite post-operative histological diagnosis whom underwent surgical procedures in China-Japan Friendship Hospital from November of 2010 to February of 2013. Age, gender, smoking history, malignancy history of patients, imaging features of the nodule including maximum diameter, position, spiculation, lobulation, calcification and serum level of CEA and Cyfra21-1 were assessed as potential risk factors. Univariate analysis model was used to establish statistical correlation between risk factors and post-operative histological diagnosis. Receiver operating characteristic (ROC) curves were drawn using different predictive models for malignant probability of SPN to get areas under the curves (AUC values), sensitivity, specificity, positive predictive values, negative predictive values for each model, respectively. The predictive effectiveness of each model was statistically assessed subsequently.

**Results:**

In 107 patients, 78 cases were malignant (72.9%), 29 cases were benign (27.1%). Statistical significant difference was found between benign and malignant group in age, maximum diameter, serum level of Cyfra21-1, spiculation, lobulation and calcification of the nodules. The AUC values were 0.786±0.053 (Mayo model), 0.682±0.060 (VA model) and 0.810±0.051 (Peking University People’s Hospital model), respectively.

**Conclusions:**

Serum level of Cyfra21-1, patient’s age, maximum diameter of the nodule, spiculation, lobulation and calcification of the nodule are independent risk factors associated with the malignant probability of SPN. Peking University People’s Hospital model is of high accuracy and clinical value for patients with SPN. Adding serum index (e.g. Cyfra21-1) into the prediction models as a new risk factor and adjusting the weight of age in the models might improve the accuracy of prediction for SPN.

## Introduction

Solitary pulmonary nodule (SPN) is defined as a spherical radiographic opacity that measures up to 3 cm in diameter and completely surrounded by lung tissue. The pathological diagnosis of SPN ranges from primary lung cancer or metastases from extrathoracic malignancy to infections, scar formation, and other benign lesions [1]. About 1 of 500 chest X-ray could display a SPN (0.2%), and more than 90% of the SPN was found without intention [2]. Surgical intervention may clarify the histological character of SPN when necessary to set up proper therapeutic strategy, and reduce the mortality associated with lung cancer [3].

## Materials and Methods

### Ethics Statement

This retrospective study was performed after been approved by the ethics committee of China-Japan Friendship Hospital, and written consent was given by the patients for their information to be stored in the hospital database and used for clinical research.

### Clinical Data

From November of 2010 to February of 2013, 107 patients with SPN confirmed by plain/enhanced chest CT scan who underwent surgical procedure in China-Japan Friendship Hospital were reviewed retrospectively. The histological result of each SPN was definite post-operatively. Based on current mathematical prediction models for malignant probability of SPN [4]–[7], clinical data including age, gender, smoking history, malignancy history, and imaging characteristics of nodule including the maximum diameter, location, spiculation, lobulation and calcification were considered as risk factors to assess (Table 1). Imaging characteristics were judged independently by two thoracic surgeons and a radiologist while the major opinion was adopted.

**Table 1:** Patient Characteristics.

Characteristic		
Gender	Male	54 (50.5%)
	Female	53 (49.5%)
Age		58.9±11.73 (24∼83)
Smoking history		40 (37.4%)*
Malignancy history		9 (8.4%)
Imaging characteristic	Maximal diameter (cm)	1.93±0.63 (0.5∼3.0)
	Located on Upper lobe	58 (54.2%)
	Spiculation	75 (70.1%)
	Lobulation	96 (89.7%)
	Calcification	7 (6.5%)

* 6 had quitted smoking for 1–20 years.

### Surgical Methods

All the patients obtained definite histological result after surgical resection.

Different surgical procedures were adopted according to the clinical diagnosis, age, heart and pulmonary function of the patients, either to the malignant probability of SPN predicted by prediction models.

1. **Wedge resection.** Linear cut stapler was applied to remove the nodule together with surrounding normal lung tissue that minimum the size of the maximum diameter of SPN. If any malignant component of SPN was explored by the frozen section, anatomical lobectomy and systematic lymphadenectomy would be performed subsequently.
2. **Segmentectomy.** Anatomical or multiple segmentectomy was performed based on the size and location of SPN, in order to keep the distance between the margin of resection and the margin of SPN not less than the maximum diameter of SPN, further steps including systematic lymphadenectomy, lobectomy or termination might be chosen based on the result of frozen section biopsy.
3. **Lobectomy.** Lobectomy might be performed directly after medical informed when the malignant probability of nodule was comparatively high, lymphadenectomy might be chosen based on the result of frozen section biopsy.

### Statistical Methods

SPSS17.0 software (2010, IBM, Chicago, US) was used for statistical analysis. The clinical data considered as risk factors associated with the malignant probability of SPN were analyzed by Univariate analysis model. Receiver operating characteristic (ROC) curves were drawn according to different mathematical prediction models. Areas under the ROC curves (AUC values) were calculated subsequently.

MedCalc12.5 software (2013, MedCalc Software Company, Acacialaan, Belgium) was used to compare the AUC values between the three different prediction models. Appropriate cut-off points considering the Youden index were determined and the sensitivity, specificity, positive predictive value, and negative predictive value were calculated.

P value<0.05 was considered statistically significant difference.

**Table 2:** Histological diagnosis and initial operation options.

Histological diagnosis		No.	Initial operation options		
			Wedge resection	Segmentectomy	Lobectomy
Benign	Inflammatory lesions	7	5	1	1
	Tuberculoma	8	5	1	2
	Aspergilloma	3	2	1	0
	Hamartoma	8	6	2	0
	Hemangioma	3	2	1	0
	Total	29 (27.1%)	20 (69.0%)	6 (20.7%)	3 (10.3%)
Malignant	Adencarcinoma	53	20	4	29
	Squamous cell carcinoma	13	4	2	7
	Small cell lung cancer	5	3	0	2
	Carcinoid	1	1	0	0
	Lymphoid epithelioma	1	1	0	0
	Sarcomatoid carcinoma	1	1	0	0
	Large cell carcinoma	1	1	0	0
	Metastatic carcinoma	3	3	0	0
	Total	78 (72.9%)	34 (43.6%)	6 (7.7%)	38 (48.7%)

## Results

### 1. Results of Post-operative Histological Diagnosis and Initial Operation Options (Table 2)

### 2. Results of Univariate Analysis

There was significant statistical difference with quantitative factors including age, maximal diameter and serum level of Cyfra21-1 between benign and malignant groups (p<0.05) (Table 3).

**Table 3:** Univariate analysis of quantitative factors.

	Benign	Malignant	p Value
Age (year)	49.2±11.74	62.4±9.56	**0.000**
Maximal diameter (cm)	1.63±0.64	2.04±0.59	**0.002**
CEA (ng/ml)	2.21±0.99	6.72±14.13	0.089*
Cyfra21-1 (ng/ml)	2.18±0.83	2.85±1.11	**0.004**

* Data of CEA did not achieve the homogeneity of variance, P value of rank-sum test >0.05, indicated no difference between groups. Result may relate to disperse distribution of value of CEA in malignant group, and could be positive after sum of case enlardged.

There were significant statistical differences with imaging characteristics including spiculation, lobulation and calcification between benign and malignant groups (p<0.05), but no statistical difference with gender, smoking history, malignancy history and location of the nodule (Table 4).

**Table 4:** Univariate analysis of qualitative factors.

		Benign	Malignant	Total	p Value
Gender	Male	17	37	54	0.209
	Female	12	41	53	
Smokinghistory	No	18	49	67	0.557
	Yes	11	29	40	
Malignancy history	No	28	70	98	0.241
	Yes	1	8	9	
Located on Upper lobe	No	15	34	49	0.297
	Yes	14	44	58	
Spiculation	No	18	14	32	**0.000**
	Yes	11	64	75	
Lobulation	No	10	1	11	**0.000**
	Yes	19	77	96	
Calcification	No	24	76	100	**0.015**
	Yes	5	2	7	

### 3. Validation and Comparison of Different Mathematical Predictive Models

According to the published literatures, the following mathematical predictive models were adopted to estimate the malignant probability while x varied by different formulas.
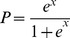



As e is the natural logarithm and qualitative factors including smoking history, malignancy history, nodule located on upper lobe, spiculation, lobulation and calcification equals 1 if exist, and 0 otherwise.

1. Mayo model:

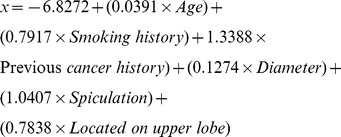

[4];

1. VA (Department of Veterans Affairs) model:

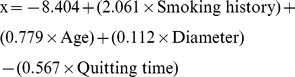

[5];

1. Peking University People’s hospital (PKUPH) model:

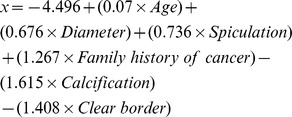
 [6], [7].

**Figure 1:**
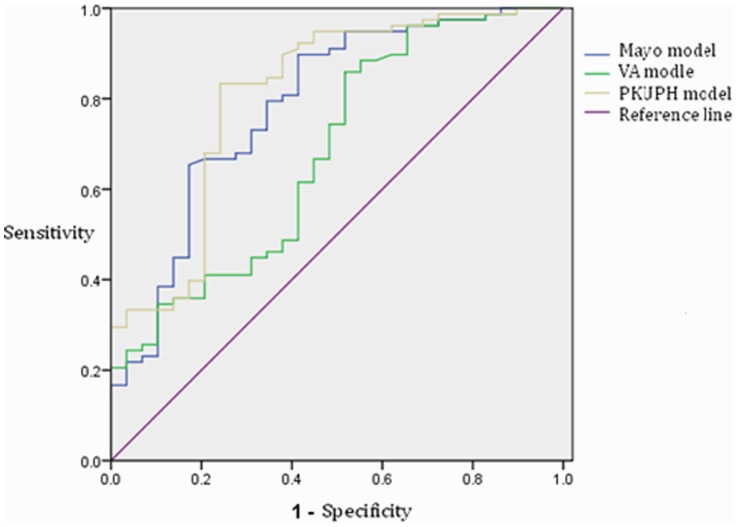
ROCs of different models.

Clinical data of 107 patients were applied to test the accuracy of different models. ROC curves were created (Figure 1) and AUC values were calculated (Table 5).

**Table 5:** Comparison of different models on AUC value*.

Models	AUC value	Standard Error	95% CI	
			Lower	Upper
Mayo model	0.786	0.053	0.683	0.889
VA model	0.682	0.060	0.565	0.799
PKUPH model	0.810	0.051	0.710	0.909

* AUC is in the range of 0.5 to 1.0. When AUC>0.5, more close to 1, higher diagnostic accuracy the model indicates. AUC In the range of 0.5∼0.7, the model has lower accuracy, 0.7∼0.9, has a certain extent of accuracy, >0.9, indicates high accuracy. When AUC = 0.5, the method shows no diagnostic value. When AUC<0.5, it does not fit the real situation.

MedCalc12.5 software was used to compare AUC values between three models. Mayo model and the Peking University people’s Hospital (PKUPH) model were proved of high accuracy, with no significant difference between each other (p = 0.577). VA model was proved of a significant lower diagnostic accuracy compared with either of other two models (p<0.05) (Table 6).

**Table 6:** Comparison of AUC values between different models.

	Mayo-VA	PKUPH-VA–	PKUPH-Mayo
Difference	0.104	0.128	0.024
Z statistic	2.504	2.764	0.558
P value	**0.012**	**0.006**	0.577

According to the ROC curves, suitable cutoff values were selected. Sensitivity, specificity, positive predictive values, negative predictive values of each model were obtained by SPSS subsequently (Table 7).

**Table 7:** Comparison of predicting performance between different models.

Models	Cutoff value	Sensitivity	Specificity	positive predictive value	negative predictive value
Mayo model	0.039	62/78(79.5%)	19/29(65.5%)	62/72 (86.1%)	19/35 (54.3%)
VA model	0.036	52/78(66.7%)	16/29(55.2%)	52/65 (80.0%)	16/42 (38.1%)
PKUPH model	0.471	65/78(83.3%)	22/29(75.9%)	65/72 (90.3%)	22/35 (62.5%)

## Discussion

Estimation of malignant probability for SPN has always been a hotspot that closely related to early diagnosis and treatment of lung cancer. Previous literatures report that age, smoking history and tumor history indicate high malignant risk of SPN [8], [9]. Image is usually needed to estimate the malignant probability of SPN, especially chest CT scan. Size and shape of nodule are most common influence factors [10], [11]. One specific independent risk factor for the malignant probability of SPN is the maximum diameter of the nodule [1]. Imaging features of SPN including density, margin and calcification are also indicated. Generally high-density solid nodule has low probability of malignancy compared with ground-glass opacity (GGO) [12]. Nodules with rough and irregular margin indicate malignancy, while calcified nodules usually tend to be benign [7], [13]. Absence of significant nodule enhancement (< or  = 15 HU) on CT scan is a strong predictive factor of benignity [14]. With active surveillance, analysis of the growth rate of nodule would be helpful to narrow the differential diagnosis, doubling time of nodule is between one month and one year would highly suggest malignancy [15]. Nowadays, PET-CT scan is proved to have an established role in the study of pulmonary nodules [16], even the estimating effect of PET-CT for nodules less than 1 cm is still controversial. The latest research also find that plasma miRNAs provide potential circulating biomarkers for noninvasively diagnosing lung cancer among individuals with SPNs [17].

However, the way to improve the level of diagnosis, staging and prognostic assessment of lung cancer with suitable cost-effect ratio is still in researching process for clinicians. Different from advanced expensive examinations and complex time-consuming follow-up mentioned above, mathematical prediction models for malignant probability of SPN provided a novel and convenient way of estimation.

Independent risk factors should be assessed before formulating the mathematical predictive model for the malignant probability of SPN. Based on previous literatures [7], [11], variables that may affect the evaluation of the malignant probability of SPN were analyzed with univariate analysis model in this study. Age of patient, maximum diameter of the nodule and imaging features including spiculation, lobulation and calcification were confirmed again as independent risk factors in our cohort.

Furthermore, serum levels of CEA (carcinoembryonic antigen) and Cyfra21-1 (cytokeratin fragment 21-1) of the malignant group were found higher than those of the benign group in this study. The difference of Cyfra21-1 between the two groups was statistical significant (p<0.05), indicating that serum level of Cyfra21-1 might be a new independent risk factor in evaluating the malignant probability of SPN.

Mayo model, VA model and PKUPH model are the three most frequently cited models during our review of literatures [5], [7], [8]. Six independent risk factors including age, smoking history, history of extrapulmonary tumors, maximum diameter and location of the nodule, as well as spiculation were confirmed in Mayo model. With good sensitivity and specificity [4], Mayo model as a model established 20 years ago, is limited from region and ethnicity, even patients with previous 5 years history of lung cancer or extrapulmonary tumors were excluded from the study inducing weakened representative. The low proportion of malignant SPN in sample of Mayo study was probably related to the definition of malignancy at that time. Furthermore, the Mayo study was also controversial since 12% of patients in this study didn’t have a definite pathological diagnosis, and considered as benign only according to no imaging change in 2-years of follow-up [5]. Independent risk factors in VA model were age, smoking history, quitting smoking period and diameter of the nodule [5]. Different from other models, risk factors in VA model did not contain imaging features, which may lead to vast deviation. Based on a retrospective study, six independent risk factors were confirmed in the predictive model of Peking University People’s Hospital, including age, maximum diameter of nodule, family tumor history, calcification, speculation and tumor margin. Different from the above mentioned models, PKUPH model adopted local influence greatly by insertion of calcification. Nodules with calcification usually tend to be benign, while the minute calcification hides malignant possibility in. The PKUPH model has high accuracy and may be more suitable for patients with SPN [6], [7].

In our validation, PKUPH model and Mayo model had higher AUC values than VA model that indicate higher diagnostic accuracy. According to our test, the sensitivity of VA model was only 66.7% and negative predictive value was less than 50%, indicating that imaging features perform great role in the evaluation of the malignant probability of SPN. In ROC curve drawn through Peking University People’s Hospital model, the cut-off point (0.471) obtained based on Youden index was similar to previous literature (0.463) [6], and sensitivity, specificity, positive and negative predictive value were all better than Mayo model, indicating the former has higher accuracy in predicting the malignant probability of SPN.

In addition, the results of specificity and negative predictive value in all three models were relatively low. Even the highest negative predictive value (Peking University People’s Hospital model) was only 62.5% (22/35), which indicate the existence of false-negative results. Hence, when the probability of malignancy of SPN evaluated by current mathematic models, it is still important to strengthen the follow-up even the predictive result is benign. For SPN predicted as malignant, it is recommended to perform a frozen section biopsy during the operation to adopt a proper subsequent surgical procedure.

13 false negative cases resulted from the PKUPH model were further investigated retrospectively. 6 cases (46.2%) were younger than 50, the youngest was only 32 years old. This result demonstrates that the sample size should be further enlarged in future to reduce the statistical bias caused by uneven distribution of age. On the other hand, it also shows that large age weights in the malignant probability model of SPN. Since air pollution and other relevant factors, age at onset of lung cancer is getting younger [18], [19]. Hence the weight of age in the malignant probability model might be adjusted for higher accuracy of prediction.

Moreover, new risk factor such as serum index (e.g.Cyfra21-1) that preliminary proved in this study might be adopted into predictive model, in order to improve the accuracy. Data of CEA in this cohort did not achieve homogeneity of variance, and no difference was found between two groups with rank-sum test (p>0.05), indicating disperse distribution of CEA in malignant group and standard deviation was huge. A positive result may be achieved if sample size could be enlarged. As a broad-spectrum tumor marker, CEA is commonly used to assess the therapeutic effectiveness of colorectal cancer, breast cancer and lung cancer, as well as index of monitor and prognosis. Also CEA has already been considered as an independent risk factor for estimating the malignant probability of SPN in literature [13]. Cyfra21-1, a soluble fragment of cytokeratin 19, is considered as one of the major tumor markers for lung cancer, especially for non-small cell lung cancer (NSCLC). Serum level of Cyfra21-1 had been preliminary proved to be an independent risk factor of malignant probability of SPN in our cohort. Kupert and colleagues also reported that the sensitivity of prediction for malignant nodules could be improved by monitoring serum level of CEA and Cyfra21-1 simultaneously [20]. These indicated that the malignant risk stratification of SPN based on previous risk factors needed to be reconsidered.

## Conclusion

In summary, PKUPH model was found to have the highest diagnostic accuracy within the three verified and compared mathematical prediction models in this study. The tendency of improving the accuracy of prediction model by adding serum index (e.g. Cyfra21-1) and adjusting the weight of age needs future prospective study. The mathematical prediction model could help to evaluate the character of SPN and set up more accurate diagnostic and therapeutic strategies.
